# Single-Molecule Imaging and Super-Resolution Microscopy of Lipid Domains in Cell Membranes Using Lipid-Binding Proteins and Fluorophore-Conjugated Lipid Analogs

**DOI:** 10.3390/membranes15100317

**Published:** 2025-10-16

**Authors:** Toshiki Mori, Kenichi G. N. Suzuki

**Affiliations:** 1 The United Graduate School of Agricultural Science, Gifu University, Gifu 501-1193, Japan; mori.toshiki.u1@s.gifu-u.ac.jp; 2 Division of Advanced Bioimaging, National Cancer Center Research Institute (NCCRI), Tokyo 104-0045, Japan; 3 Institute for Glyco-Core Research (iGCORE), Gifu University, Gifu 501-1193, Japan

**Keywords:** lipid probes, lipid-binding proteins, single-molecule observation, super-resolution microscopy

## Abstract

Lipids are spatiotemporally organized in cell membranes, where they play indispensable roles in regulating diverse biological processes. Their distribution and dynamics are intricately coupled to signal transduction, membrane trafficking, and host–pathogen interactions. The past decade has seen substantial progress in the development of lipid probes and imaging techniques, which have greatly advanced our understanding of lipid-mediated regulation in living cells. Chemically optimized lipid analogs conjugated with hydrophilic fluorophores have enabled the faithful visualization of raftophilic lipids, such as sphingomyelin, gangliosides, and cholesterol, while minimizing artifacts. In parallel, genetically encoded lipid sensors derived from lipid-binding protein domains have been established. These sensors selectively report the localization and dynamics of diverse lipid species, including phosphoinositides, cholesterol, sphingomyelin, and phosphatidylserine, in their native contexts. Combined with state-of-the-art advanced microscopy approaches, including ultrafast single-molecule imaging and super-resolution microscopy, these probes facilitate high-resolution and quantitative analyses of lipid organization. This review summarizes recent advances in both synthetic lipid probes and genetically encoded lipid sensors, emphasizing their applications in mechanistic studies of membrane biology. We further discuss current challenges and future directions toward the comprehensive and minimally perturbative visualization of lipids.

## 1. Introduction

The spatial and temporal organization of lipids in cell membranes plays a pivotal role in diverse cellular processes, including signal transduction, membrane trafficking, pathogen entry, and intercellular communication [1,2]. Membrane domains in distinct lipid species, including phosphoinositides, sphingolipids, gangliosides, and cholesterol, serve as dynamic platforms that recruit and orchestrate signaling proteins, modulate membrane curvature, and regulate vesicular transport. Therefore, elucidating the distribution and rapid remodeling of these lipid assemblies is indispensable for uncovering the fundamental mechanisms underlying a broad spectrum of biological events, such as immune responses [3,4], neuronal signaling [5,6], cancer progression [7,8], and host–pathogen interactions [9,10].

Traditionally, the fluorescence imaging of lipids has relied on antibodies or lipid-binding toxins applied following chemical fixation [11,12]. Fixation with formaldehyde and/or glutaraldehyde efficiently immobilizes membrane proteins by crosslinking amino acids, hydroxy groups, and thiols, but fails to covalently crosslink most lipid species, thereby permitting substantial lateral diffusion, extraction, or redistribution before immobilization [11,12]. To label lipids in the inner leaflet of plasma membranes (PMs), fixed cells must be permeabilized with detergents; however, detergent permeabilization further perturbs the membrane architecture by solubilizing specific lipid populations and disrupting discrete membrane domains. In addition, fixation can alter the lateral distribution of lipids and proteins through protein crosslinking [13,14,15]. Furthermore, the binding of multivalent antibodies or toxins (e.g., cholera toxin B subunit for GM1) can artificially cluster lipids and induce non-physiological membrane rearrangements [12]. Such artifacts obscure the native lipid distribution and may yield erroneous interpretations of their biological functions.

Because the lipid domains in cell PMs reorganize on sub-second timescales in response to environmental cues [16,17], receptor activation [18,19], or mechanical stress [20], only live-cell imaging can faithfully capture their native distribution and dynamic remodeling [21,22]. Recent advances in minimally perturbative lipid probes, tagged with organic fluorophores and biosensors, including lipid-binding proteins, enable the direct visualization of specific lipid distributions in both the outer and inner leaflets of cell PMs. Moreover, state-of-the-art imaging modalities, such as ultrafast single-molecule tracking [23] and super-resolution microscopy [24,25], facilitate the visualization of lipid domains with spatial resolutions of 10–20 nanometers and temporal resolutions of approximately 30 microseconds in living cells. These approaches provide critical insights into the dynamic properties of lipids, including how lipid heterogeneity governs protein recruitment, signaling complex assembly, and membrane remodeling, thereby bridging the gap between lipid biochemistry and cellular physiology.

In this review, we comprehensively highlight studies employing lipid probes, including lipids conjugated with organic fluorophores and lipid-binding proteins. Furthermore, we introduce advanced microscopic techniques, such as high-speed single-molecule imaging and super-resolution microscopy, which are utilized to visualize the lipid distribution in cell PMs.

## 2. Observation of Lipid Probes Conjugated with Organic Dyes in the Outer Leaflet of the PM in Living Cells

### 2.1. Development of Lipid Probes Conjugated with Organic Dyes

Lipid probes conjugated with organic fluorophores represent a powerful approach for investigating the spatial distribution and dynamic behavior of lipids in the outer leaflet of the PM. Numerous dye-conjugated lipids, including derivatives of sphingolipids and phosphatidylcholine (Table 1), when suspended in observation media such as HBSS, can be incorporated into the outer leaflet of PMs [26,27]. This strategy enables the live-cell imaging of lipid organization, domain formation, and trafficking at high spatial and temporal resolutions using advanced fluorescence microscopy, while preserving the physiological orientation and accessibility of the target lipid species.

Phospholipids conjugated with organic dyes are frequently generated by covalently attaching hydrophilic fluorophores to the polar headgroups of phospholipids, most commonly via the primary amine of phosphatidylethanolamine (PE). The ethanolamine moiety provides a reactive site for the formation of amide- or carbamate-linked conjugates with N-hydroxysuccinimide (NHS)-activated esters or isothiocyanate derivatives of fluorophores such as fluorescein [28], tetramethylrhodamine [29], Alexa [36], Cy3 [23], or ATTO [30] dyes (Table 1). This headgroup-specific conjugation preserves the hydrophobicity and bilayer-inserting capacity of the diacylglycerol backbone while exposing the fluorophore to the aqueous phase, thereby minimizing interference with the lipid’s membrane anchoring. An alternative strategy involves the covalent attachment of hydrophobic fluorophores, such as BODIPY-FL, BODIPY-TR, or NBD, to the acyl chains of phospholipids. In this approach, the fluorophore is introduced at a defined position along the saturated or unsaturated fatty acyl chain via an amide or ester linkage, yielding analogs such as BODIPY-FL-C5- or -C12-phosphatidylcholine (PC) and -phosphatidylethanolamine (PE) (Table 1) [32,33]. The placement of the dye within the hydrophobic tail anchors it deep in the bilayer interior, closely mimicking the natural insertion of fatty acyl chains. The hydrophobic nature of the conjugates facilitates rapid and spontaneous incorporation into membranes.

### 2.2. Single-Molecule Tracking of Phospholipid Diffusion at High Temporal Resolution

Single-molecule tracking enables direct measurement of the trajectories of individual lipid molecules, from which diffusion coefficients and confinement properties can be derived. In particular, the application of total internal reflection fluorescence microscopy (TIRFM) with high-speed cameras has allowed the detailed characterization of lipid diffusion. Using single-molecule tracking at a 100 μs temporal resolution, Fujiwara et al. demonstrated that Cy3-dioleoylphosphatidylethnolamine (Cy3-DOPE, Table 1) underwent confinement in small domains (~100 nm), intermittently hopping to adjacent domains with a lifetime of ~10 ms, and repeatedly exhibiting this behavior in T24 cells [23]. This diffusional mode, termed “hop diffusion”, can be revealed only by single-molecule imaging at ultrahigh temporal resolution. In contrast, Cy3-DOPE displayed simple Brownian diffusion on membrane blebs devoid of cortical actin filaments. Notably, the compartment size of Cy3-DOPE was comparable to that observed for the transferrin receptor (TfR), although the residency time of TfR within compartments (24 ms) was longer than that of Cy3-DOPE. Based on these findings, Fujiwara et al. proposed the “fence and picket” model to explain hop diffusion. In this model, the actin-based membrane skeleton underlying the PM establishes the fundamental compartment boundaries, as molecules collide with the actin meshwork on the inner leaflet of the PM. Furthermore, transmembrane “picket” proteins, anchored to and aligned along the actin-based fence, act as diffusion barriers for molecules residing in the PM. The actin-based cytoskeleton thereby attenuates inter-compartmental movement through steric hindrance. Intriguingly, even the phospholipids located in the outer leaflet of the PM exhibited hop diffusion, with the compartment size distributions resembling the mesh size of the actin-based cytoskeleton. Monte Carlo simulations suggested that even if transmembrane picket proteins occupy only 17% of the compartment boundary, their immobility generates a hydrodynamic friction-like effect sufficient to restrict molecular diffusion [47].

Cy3 is the most suitable organic fluorophore covalently conjugated to phospholipids for high-speed single-molecule observation at a 100 μs resolution, as it exhibits minimal blinking, and the number of photons emitted from Cy3 increases proportionally with elevated laser illumination intensity [23]. Although the quantum yield of Cy3 in observation media is below 0.2, its fluorescence lifetime is short (0.3–0.4 ns) due to its propensity for cis-trans isomerization [48], and the photon output is sufficient to generate the distinct fluorescent spot even at acquisition rates exceeding 1 kHz [23].

### 2.3. Development of Raftophilic Lipid Probes Tagged with Organic Fluorophores

**Figure 1:**
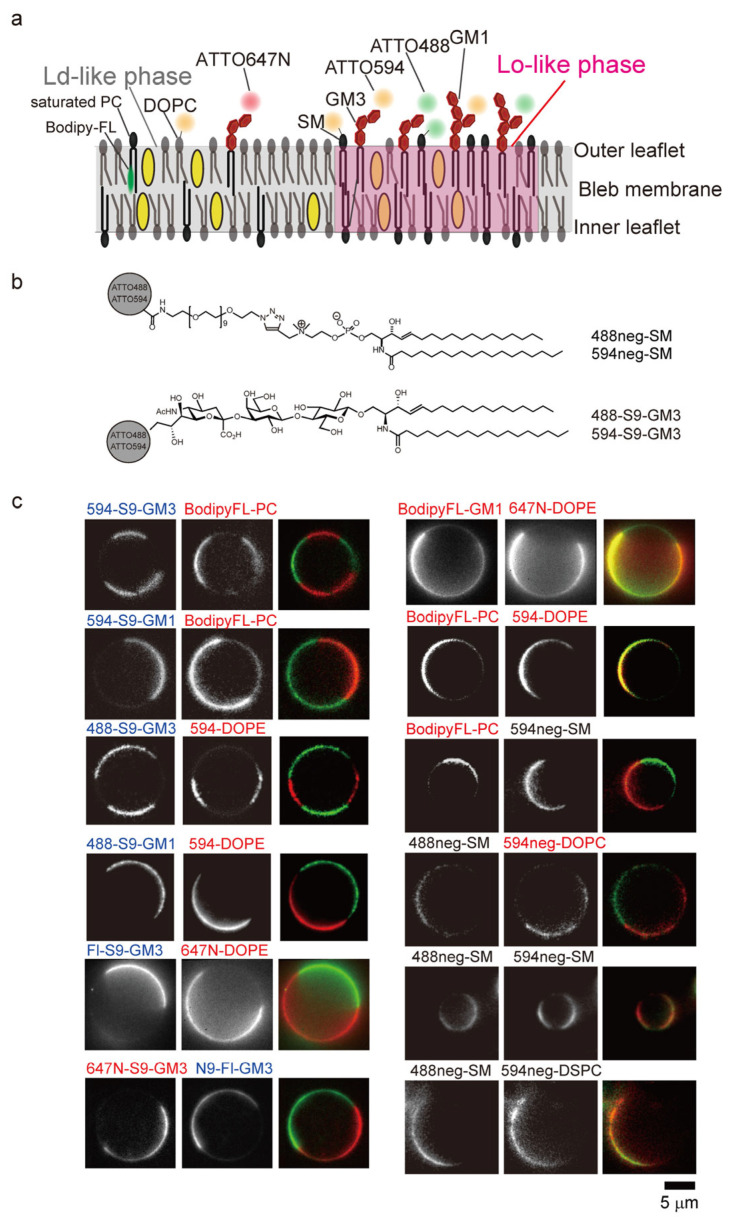
Raftophilic lipid probes conjugated with fluorescent organic dyes. (**a**). Schematic diagram of the partitioning of lipid probes into L_o_ (raft)-like (magenta) and L_d_ (non-raft)-like domains (gray). GM3 or SM probes conjugated with hydrophilic dyes at the sialic acid via nonaethyleneglycol (neg) preferentially partitioned into L_o_-like phase, whereas probes conjugated with hydrophobic dyes such as ATTO647N partitioned into L_d_-like phase [27,34]. (**b**). Chemical structures of 488neg-SM and 594neg-SM [26] (**top**), and 488-S9-GM3 and 594-S9-GM3 [27] (**bottom**). (**c**). Representative fluorescence images from simultaneous dual-color video sequences of the probes in membrane blebs of RBL-2H3 cells at 8 °C [26,27]. Probes in blue and red denote those localized in raft-like and non-raft-like phases, respectively. “S9” indicates that fluorescent dyes were conjugated to the C9 position of the terminal sialic acid of GM3 or GM1 [27]. “Neg” denotes that nonaethyleneglycol was used as a spacer between the choline moiety of SM or PC and the dyes [26]. Adapted from Figure 1, 5 in [26] and Figure 2 in [27] with permission.

Lipid probes tagged with organic fluorophores have also been employed in studies of lipid rafts in cell PMs. Probes for raftophilic lipids such as sphingomyelin (SM), glycosphingolipid, phosphatidylcholine, and phosphatidylethanolamine containing two saturated fatty acids and cholesterol have been developed and examined in the outer leaflet of cell PMs (Figure 1a).

SM, a major sphingolipid, is a prototypical raftophilic lipid that participates in diverse cellular processes, including autophagy, apoptosis, and inflammation. Structurally, SM consists of a sphingosine backbone, a fatty acid, and a phosphocholine or phosphoethanolamine headgroup, and is unique among phospholipids in lacking a glycerol moiety. The amide and hydroxyl groups of the sphingosine backbone establish an intermolecular hydrogen-bonding network, which is further stabilized by cholesterol. Several SM probes conjugated with organic fluorophores have been developed, in which dyes were linked to short fatty acids (C5 or C12) [49] or to the ethanolamine group of ceramide phosphoethanolamine [30] (Figure 1a). However, likely due to the steric hindrance introduced by bulky dyes and the strong hydrophobicity of conjugated fluorophores, these SM probes failed to recapitulate the behavior of native SM molecules. Instead, the majority partitioned into the liquid-disordered (Ld) phase of giant unilamellar vesicles (GUVs) and giant plasma membrane vesicles (GPMVs) [34].

To preserve the raftophilic properties of SM, namely, its preferential partitioning into the liquid-ordered (Lo) phase in GUVs and GPMVs, as well as into detergent-resistant membrane (DRM) fractions, Kinoshita et al. developed optimized SM probes [26]. In these probes, hydrophilic fluorophores such as ATTO488 and ATTO594 were conjugated to the choline headgroup of SM via a hydrophilic nonaneethylene glycol (neg) linker, using a click reaction that retained the positive charge of the headgroup (Figure 1b, top). These probes, termed 488neg-SM and 594neg-SM, preferentially partitioned into the Lo-phase in GUVs and Lo-like phase in GPMVs [26] (Figure 1c and Table 1). Additionally, ATTO594-neg-distearoylphosphatidylcholine (594neg-DSPC) and ATTO594-neg-dioleoylphosphatidylcholine (594-DOPC) were developed, which preferentially localized to the Lo and Ld phases, respectively [26] (Figure 1c and Table 1). Another group synthesized a PE probe by conjugating distearoylphosphatidylethanolamine (DSPE) with a hydrophilic fluorophore (KK114) via a very large hydrophilic linker, a 2 kDa polyethylene glycol (PEG) linker, which exhibited preferential partitioning into the Lo phase (Table 1) [31].

Gangliosides are glycosphingolipids containing one or more sialic acid residues and are regarded as prototypical raftophilic lipid markers. Glycosphingolipids, including gangliosides, play pivotal roles in numerous physiological processes, such as regulating receptor activity [50]; mediating the cellular invasion of microbial toxins, viruses, and bacteria [51]; and contributing to the pathogenesis of disorders including Guillain-Barré syndrome [52], Alzheimer’s disease [53], and Parkinson’s disease [51]. The cholera toxin subunit B (CTXB) has been most widely used as a marker for GM1, the predominant ganglioside [54]. However, CTXB forms a pentamer that crosslinks five GM1 molecules, thereby altering their native localization. Thus, glycosphingolipid probes, including gangliosides that faithfully mimic the behavior of their parental counterparts, are highly warranted. To date, a variety of fluorescent glycolipid probes that partition into Lo-like phases in GPMVs have been synthesized (Table 1). Lencer’s group developed fluorescent GM1 analogs with C18:0, C16:0, C12:0, or C16:1 fatty acyl chains [36], in which Alexa-568 or Alexa-488 was conjugated to the C7 position of the sialic acid (Table 1). They reported that the CTXB binding affinity of Alexa568-GM1 was approximately 10-fold lower than that of native GM1, whereas Alexa488-peptide-GM1 exhibited binding comparable to that of the parental molecule [37]. Similarly, Ando’s group synthesized diverse fluorescent glycolipid probes that preferentially partition into the Lo-like phases in GPMVs [27,35]. They synthesized fluorescent analogs of GM1, GM2, GM3 (Figure 1b, bottom) and GD1b by labeling the parental gangliosides with fluorescein, ATTO488, and ATTO594 at specific positions: the C9 position of the terminal sialic acid (S9-type for GM1, GM2, and GM3), the C6 position of the terminal galactose (G6-type for GM1 and GD1b), and the C6 position of the terminal GalNAc (GN6-type for GM2), using a method entirely based on chemical synthesis (Table 1) [27]. All of these ganglioside probes partitioned into the DRM fractions and Lo-like phases in GPMVs (Figure 1c). Surface plasmon resonance analyses demonstrated that GM1 and GM3 probes bound to CTXB and wheat germ agglutinin (WGA) with affinities equivalent to those of their parental counterparts [27]. In contrast, when the C9 position of the sialic acid was conjugated with more hydrophobic fluorophores such as ATTO647N or tetramethylrhodamine (TMR), the probes failed to preferentially partition into Lo-like phases in GPMVs (Figure 1c) [27]. Similarly, an additional series of ganglioside probes, including lacto-series and globo-series gangliosides that partitioned into the Lo-like phase (Table 1), have also been developed [35]. These findings suggest that the conjugation of hydrophilic fluorophores to terminal sugar residues generally preserves raftophilic behavior.

Numerous cholesterol probes conjugated with organic fluorophores, including NBD and BODIPY-FL, have also been developed (Table 1). However, most failed to partition into the Lo-phase of GUVs (Figure 1c). The exception is a cholesterol probe conjugated with BODIPY-FL at carbon 24 of the alkyl side chain via the central dipyrrometheneboron difluoride ring [42,43].

### 2.4. Microscopic Observation in the Outer Leaflet of the PM

SM, ganglioside, saturated PC, and cholesterol probes can be incorporated into cell PMs, and their dynamic behaviors have been investigated at high temporal resolution. Transient immobilization of these raftophilic lipid probes has frequently been observed. For example, studies using stimulated emission depletion (STED) microscopy combined with fluorescence correlation spectroscopy (FCS) demonstrated that a GM1 probe conjugated with ATTO647N at either the sugar moiety and an SM probe labeled with ATTO647N at either the headgroup or the fatty acyl chain frequently underwent temporal confinement in small membrane regions (<20 nm in diameter) for approximately 10 ms in a cholesterol-dependent manner. Between confinement events, these probes diffused freely at ~0.5 μm^2^/s [30]. The fractions of time spent by GM1 and SM probes in these confined regions ranged from 45 to 60% [30]. These findings have been corroborated by several other studies [55,56]. However, because these GM1 and SM probes partitioned almost entirely into the L_d_ phase in model membranes [34], these results may not necessarily reflect transient trapping in nanoscale raft domains.

The single-molecule tracking of GM1 and GM3 probes conjugated with ATTO594 at the C9 position (594-GM1 and 594-GM3) at a 0.5 ms resolution revealed that these ganglioside probes were rarely confined in small domains (~100 nm in diameter) for durations longer than 5 ms in four types of cell PMs [27]. Since these GM1 and GM3 probes partition into the Lo-like phase in GPMVs [27] and are thus considered reliable raft markers, these results suggest that ganglioside probes are seldom trapped in raft domains ~100 nm in diameter for longer than 5 ms in the examined cell types (PtK2, T24, NRK, and COS7 cells) [27].

Furthermore, single-molecule tracking at a 0.5 ms resolution revealed that neither raftophilic lipid probes such as 594neg-SM and 594neg-DSPC nor a non-raftophilic lipid probe, 594neg-DOPC [26], were transiently confined in small raft domains ~100 nm in diameter for durations longer than 5 ms in two cell types (T24 and PtK2). Because 594-negSM and 594-negDSPC partitioned into the Lo-like phases in GPMVs, together with the aforementioned observations for ganglioside probes [26], these findings indicate that gangliosides, SM, and saturated PC probes do not undergo temporal confinement in 100 nm domains for more than 5 ms. To our knowledge, there have been no reports of epi-fluorescence microscopy observations showing that lipid probes conjugated with organic dyes are extensively enriched in domains such as caveolae or flotillin in the steady-state cell PMs, which is consistent with the results of single-molecule observations.

**Figure 2:**
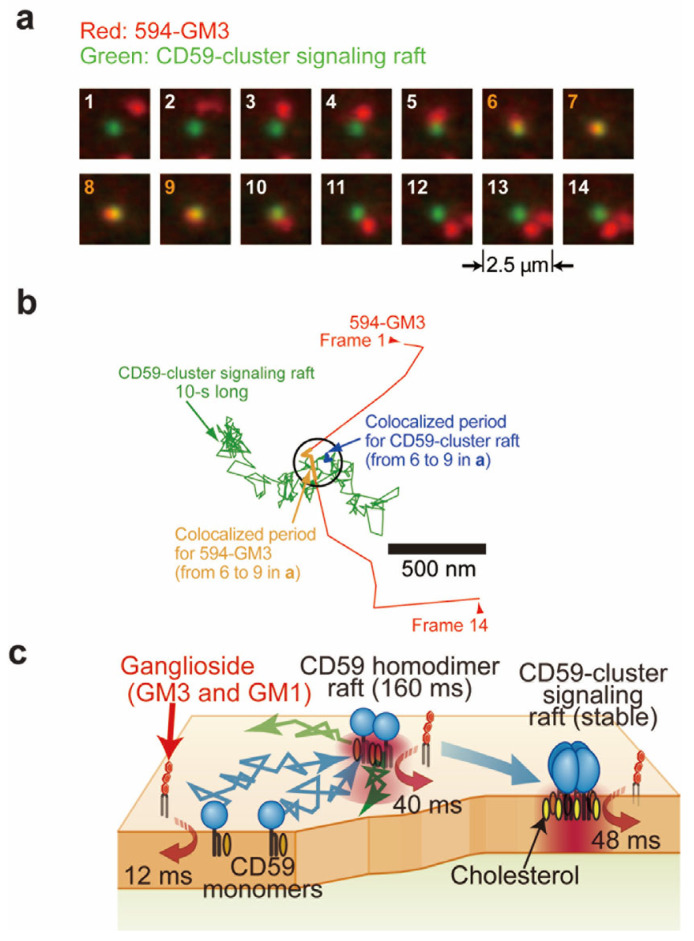
Recruitment of a ganglioside probe (GM3) to raft domains in cell PMs. (**a**). Representative image sequence of CD59 cluster-signaling rafts (green) and a single molecule of ATTO594-conjugated GM3 (red) in the T24 cell PM at 37 °C, which were recorded at video rate [27]. (**b**). Trajectories of CD59 cluster rafts and ATTO594-conjugated GM3 shown in (**a**) [27]. (**c**). Schematic diagram of the recruitment of ganglioside probes to CD59. Single molecules of ganglioside probes were frequently and transiently recruited to CD59 monomers, homodimers in steady state, and CD59 clusters upon stimulation, with characteristic timescales of 12, 40, and 48 ms, respectively [27]. Adapted from Figure 5 in [27] with permission.

Meanwhile, single molecules of 594-GM1 and 594-GM3 were transiently recruited to glycosylphosphatidylinositol (GPI)-anchored protein, and CD59 monomers and homodimers [57] for 12 and 40 ms, respectively [27] (Figure 2a–c). Moreover, 594-GM1 and 594-GM3 were recruited to CD59 clusters, which initiate intracellular signaling [58,59], for prolonged durations of 48 ms [27] (Figure 2a–c). Similarly, single molecules of 594neg-SM were recruited to CD59 monomers, homodimers, and clusters for 12, 33, and 50 ms, respectively [26]. These results indicate that ganglioside and sphingomyelin probes are preferentially confined in the ligand-induced small dynamic raft assemblies.

Consistent with these results, fluorescence anisotropy imaging by Arumugam et al. demonstrated that Alexa488-peptide-GM1 with a saturated C16:0 chain clustered into nanodomains through an actin-dependent process requiring cholesterol and phosphatidylserine, whereas the unsaturated C16:1 GM1 probe did not [37]. CTXB binding expanded domains in both species; however, the ceramide structure continued to modulate their organization. Co-clustering with CD59 occurred exclusively for saturated GM1 [37]. These results highlight that ceramide saturation is a critical determinant of GM1 nanodomain formation, thereby influencing membrane organization.

Single-molecule tracking of a fluorescent cholesterol analog, BODIPY488-conjugated cholesterol (Bdp-Chol), revealed that Bdp-Chol displayed an exceptionally high diffusion coefficient of 3.4 μm^2^/s. This value is the highest reported for any membrane molecule and approximately 10-fold greater than that of non-raft phospholipids (~0.33 μm^2^/s) [44]. Bdp-Chol exhibited no confinement over timescales exceeding 0.5 ms [44], similar to ganglioside and SM probes. In actin-depleted blebbed membranes, its diffusion coefficient (~5.8 μm^2^/s) was comparable to that of a phospholipid probe (Cy3-DOPE, ~6.2 μm^2^/s) [44], indicating that the cortical actin skeleton reduced Bdp-Chol diffusion by only ~twofold, in contrast to the ~20-fold reduction observed for phospholipids. These observations suggest that cholesterol can transverse membrane compartments more readily than phospholipids and that lipid raft domains coexist within actin-defined compartments. In addition, the larger diffusion coefficients of Bdp-Chol than a non-raft phospholipid can also be explained by free area theory [60].

Lipid probes conjugated with spontaneously blinking organic fluorophores, such as SF650B (identical to HMSiR), are valuable for super-resolution dSTORM imaging. For example, Hirosawa et al. incorporated GM3 conjugated with SF650B at the C9 of the terminal sialic acid into liposomes, and performed dSTORM imaging by observing single molecules at a 4 ms resolution for 10,000 frames [24]. This approach enabled the precise determination of liposome diameters, which were highly consistent with those measured by electron microscopy or the electrical resistance nanopulse method [24]. However, it should be noted that, due to the moderate hydrophobicity of SF650B, the SF650B-GM3 probe partitioned into the cold 1% Triton X-100-soluble (non-raft-like) fraction, and therefore cannot serve as a reliable raft marker.

## 3. Observation of Lipids in the Inner Leaflet of Cell PMs Using Lipid-Binding Proteins

Because lipid probes tagged with organic dyes cannot be incorporated into the inner leaflet of cell PMs without fixation and permeabilization, alternative strategies are required. To investigate the localization and dynamics of lipids in the inner leaflet of living cells, lipid-binding proteins expressed in the cytosol of cells represent a highly effective tool. When fused to fluorophores via tags, these lipid-binding proteins enable the visualization of lipid distribution and dynamics in the inner leaflet or intracellular compartments using confocal microscopy [61,62], single-molecule imaging [63,64], and super-resolution microscopy [65,66,67] (Table 2). In this section, we summarize recent advances in the development of lipid-binding protein probes and their applications in state-of-the-art imaging methodologies.

### 3.1. Development of Phosphoinositide Probes Using Lipid-Binding Proteins

Because phosphoinositides are predominantly localized to the inner leaflet of cell PMs, it may not be easy to use phosphoinositides conjugated with organic dyes as probes. Instead, genetically encoded fluorescent biosensors, generated by fusing phosphoinositide-binding domains from effector proteins to fluorescent tags, have proven to be highly effective tools for monitoring lipid dynamics in living cells.

As a genetically encoded sensor for PI(4,5)P_2_, the PH domain of PLCδ1 fused to fluorescent proteins (e.g., GFP) has long been widely used, enabling the live-cell imaging of membrane localization [61,62,65,68,69]. More recently, live-cell studies have demonstrated that the C-terminal domain of Tubby (tubbyCT) accumulates at ER-PM junctions via coincidence detection with extended synaptotagmin 3, thereby allowing selective readouts of compartmentalized PI(4,5)P_2_ at contact sites [70,82]. TubbyCT serves as a novel selective reporter for ER-PM junctional pools of PI(4,5)P_2_. Furthermore, tandem or multimerized tubbyCT constructs enhance the apparent affinity and signal-to-noise ratio, improving the detection of subtle PI(4,5)P_2_ fluctuations at both the PM and ER-PM junctions. More recent innovations include cell-permeable fluorescent peptide probes derived from actin-binding fragments (e.g., N-terminus 12- or 20-amino-acid peptides of gelsolin), which selectively detect PI(4,5)P_2_ dynamics in living cells with high sensitivity and temporal resolution on the order of seconds [114].

The PH domain of Akt functions as a classical biosensor for PI(3,4,5)P_3_ [86,87,88]. The PH domain of the general receptor for phosphoinositides isoform 1 (GRP1), a PI3K-activated exchange factor for Arf GTPases, selectively binds to PI(3,4,5)P_3_ with high affinity and has been widely used as a marker [89]. Upon PI3K activation, the Akt-PH domain translocates to the PM, reflecting PI(3,4,5)P_3_ accumulation and pathway activation. PI(3,4,5)P_3_ regulation is determined by amino acid residues distributed across the PH domain, rather than solely the PIP_3_-binding pocket [87].

Dowler et al. [115] identified tandem-PH-domain containing protein 1 (TAPP1) as the first protein reported to selectively bind phosphatidylinositol-3,4-biphosphate (PI(3,4)P_2_) over other phosphoinositides. The C-terminal PH domain of TAPP1 was initially employed to visualize PI(3,4)P_2_ [83], although the sensitivity of the single domain was limited. Enhanced specificity and signal strength were subsequently achieved using tandem or trimer constructs, which reliably bound PI(3,4)P_2_ at the PM with high avidity and selectivity [71].

More recently, Vines et al. characterized Dictyostelium SnxA as a highly selective PI(3,5)P_2_-binding protein and validated GFP-SnxA as a PI(3,5)P_2_ reporter in mammalian cells [84]. Upon paraformaldehyde fixation, GFP-SnxA exhibited partial enrichment near the plasma membrane. The PX domain of SnxA constitutes a highly selective PI(3,5)P_2_-binding domain module; however, a single PX-GFP fusion protein displayed minimal affinity for PI(3,5)P_2_ [84]. Although tandem repeats of the PX domain enhanced binding affinity, the affinity remained lower than that of the full-length SnxA. Furthermore, Rizalar et al. demonstrated that the PH domain of the kinesin KIF1A directly binds to PI(3,5)P_2_ and, to a lesser extent, to PI(3)P in vitro [85].

Lipid phosphatidylinositol 4-phosphate (PI(4)P) probes have been engineered by fusing lipid-binding domains, such as four-phosphate-adaptor protein 1 (FAPP1) or oxysterol-binding protein homolog (OSH) proteins, with fluorescent tags to visualize localization at the Golgi and PMs [92]. The PH domain of the PI(4)P-binding protein SidM (P4M) is not biased by interactions with Arf1 [72]. These probes enable the detailed investigation of PI(4)P-mediated trafficking and lipid exchange between membranes.

The Fab1, YGL023, Vps27, EEA1 (FYVE) domain comprises a small Zn^2+^-binding module of 60–70 amino acids enriched in cysteine residues. When employed as tandem repeats (e.g., 2xFYVE), the FYVE domain binds to PI(3)P with markedly greater affinity than single FYVE fingers [93,94,95], effectively labeling early endosomal membranes in cells. Structural and mechanistic studies have validated FYVE domain’s high specificity for PI(3)P and elucidated the molecular basis of this interaction, which involves electrostatic binding, hydrophobic insertion, and a protonation-dependent “histidine switch” [96].

### 3.2. Development of Phosphatidylserine Probes in Cell Membranes

Phosphatidylserine (PS) is a major anionic phospholipid predominantly localized to the cytosolic leaflet of the cell PM. The development of genetically encoded probes derived from PS-binding protein domains has enabled the real-time visualization of PS dynamics in living cells with high specificity.

The C2 domain of lactadherin (Lact-C2), a glycoprotein originally identified in milk fat globule membranes, binds to PS with high affinity in a Ca^2+^-independent manner. Yeung et al. [98] demonstrated that EGFP-tagged Lact-C2 specifically localizes to the inner leaflet of the cell PM in a PS-dependent manner, as confirmed by the genetic depletion or enzymatic conversion of PS. The lack of Ca^2+^ dependence distinguishes Lact-C2 from annexin-based probes, reducing interference from Ca^2+^ signaling. Tandem constructs (2xLact-C2) have been generated to enhance membrane association and signal stability, establishing Lact-C2 as a widely adopted standard for PS imaging across diverse microscopy platforms [99,100,101].

Evectin-2 is a membrane-associated protein localized to recycling endosomes, where it contributes to vesicular trafficking. Its PH domain specifically binds to PS in the cytosolic leaflet of endosomal membranes. Uchida et al. [102] first identified this property through liposome-binding assays and mutagenesis, demonstrating that conserved basic residues within the PH domain mediate direct PS recognition. When fused to fluorescent proteins, the evectin-2 PH domain functions as a genetically encoded biosensor for visualizing intracellular PS pools in live cells. Notably, the evectin-2 PH probe exhibited negligible binding to phosphoinositides such as PI(4,5)P_2_, thereby reducing cross-reactivity and enhancing accuracy in complex lipid environments. The evectin-2 PH domain has also been used as a sensor for PS in the inner leaflet of cell PMs [65,106].

### 3.3. Development of SM-Binding Protein Probes in Cell Membranes

Several SM-binding protein probes have been developed. Lysenin [110,116] and members of the actinoporin family, such as equinatoxin II (EqtII) [117] from the sea anemone *Actinia equina*, are pore-forming toxins with intrinsic SM-binding activity. Lysenin consists of an N-terminal pore-forming domain comprising the first 160 amino acids, while its C-terminal region (residues 161–297), referred to as NT-lysenin, harbors the SM-binding site but lacks the ability to homo-oligomerize or form pores [111]. Notably, full-length lysenin carrying the W20A mutation is also unable to oligomerize [113]. The interaction of lysenin with SM is strongly influenced by the local distribution and density of the lipid. Fluorescently labeled NT-lysenin and W20A-lysenin have been employed to visualize SM in the plasma membrane (PM) [66,113].

Similarly to lysenin, EqtII is cytotoxic; however, the L26A/P81A double mutant abolishes its pore-forming activity while preserving the wild-type’s specificity and affinity for SM. The truncated form, NT-EqtII, has been successfully applied to monitor the SM distribution in the PMs of living cells [65]. Lysenin preferentially binds to SM clusters, whereas EqtII recognizes more dispersed SM [118].

### 3.4. Development of Cholesterol-Binding Protein Probes in Cell Membranes

Various protein probes have been developed for visualizing cholesterol in cell membranes. Iwashita’s group first identified perfringolisin O (PFO, also known as theta toxin), a cytolysin produced by Gram-positive bacteria, which preferentially binds to cholesterol in membrane domains enriched in both cholesterol and stearoyl chains [119,120,121]. The theta toxin comprises four domains (D1–D4) and forms oligomeric pore complexes in membranes, with the D4 domain representing the minimal fragment required for cholesterol binding [120]. Fusion of the D4 domain to a fluorescent protein (e.g., mCherry) enabled imaging of the cholesterol distribution in intact erythrocyte membranes [122]. A point mutation in D4 (D434S), termed D4H, was subsequently engineered to enhance cholesterol-binding affinity [123] and was used to demonstrate that the cholesterol content in the inner leaflet of CHO-K1 cell PMs is lower than that in the outer leaflet [73]. Together with other reports [124], these results suggest that the inner leaflet of CHO-K1 cell PM contains less cholesterol than that of HeLa or MA-10 cells.

Kemmoku et al. [107] further reported that STING clusters formed in the trans-Golgi network (TGN) were strongly colocalized with mNeonGreen-iD4H, indicating that STING clusters associate with cholesterol-enriched domains in the TGN. In addition to mammalian systems, Fernández-Golbano et al. [108] developed a live-cell imaging protocol for monitoring ergosterol dynamics in *Saccharomyces cerevisiae*, enabling the temporal analysis of sterol distribution under conditions of biosynthesis inhibition. In tissue applications, de Leeuw and Nuriel [109] established a histological approach using high-affinity D4H-mCherry to visualize intracellular cholesterol in mouse and human brain sections. Co-staining with endosomal markers provided precise localization within subcellular compartments. Collectively, these advances extend the applicability of genetically encoded cholesterol sensors from unicellular to complex tissues, facilitating spatiotemporal mapping of sterol dynamics across diverse biological contexts.

## 4. Advanced Microscopic Observation of Lipid-Binding Protein Probes in Cells

To obtain deeper insights into the dynamics and spatial distribution of lipids in the inner leaflet of the cell PM, high-speed single-molecule fluorescence imaging and super-resolution microscopy of lipid-binding protein probes represent highly powerful methodologies, as described in the section on organic dye-conjugated lipid probes in the outer leaflet of the cell PM. In this section, we introduce studies on lipid domain architectures and the interactions between lipids and membrane proteins using lipid-binding protein probes with these advanced microscopic approaches.

### 4.1. Observation in Fixed Cells

In many cases, super-resolution microscopy of lipid-binding proteins has been performed after chemical fixation. Mizuno et al. [112] expressed the recombinant protein domains, D4 of θ-toxin and NT-lysenin in *E. coli* as fusions with the photoswitchable fluorescent protein Dronpa. These probes were applied to fixed HeLa cells, where PALM was subsequently performed. They demonstrated that both D4 and NT-lysenin were organized into clusters with diameters larger than 200 nm. Abe et al. [66] further reported that the overexpression of peripheral myelin protein 2 (PMP2) in HeLa or MDCK cells increased the proportion of SM in the inner leaflet while concomitantly decreasing that in the outer leaflet. They performed super-resolution STORM imaging of single Halo-NT-lysenin molecules conjugated with the spontaneously blinking dye SF650B in fixed cells to evaluate the abundance of SM domains in the inner leaflet. Abe et al. [67] also showed that EGFR activation reduced its colocalization with PI(4,5)P_2_ due to PI(4,5)P_2_ hydrolysis by PLCγ, as revealed by dual-color super-resolution PALM and dSTORM analyses of EGFR together with PS, PI(4,5)P_2_, or PI(3,4,5)P_3_ in fixed HeLa or CHO cells. Wang et al. [74] conducted dSTORM imaging of EGFR labeled with Alexa647-cetuximab in normal lung epithelial cells and lung cancer cells, and observed that the EGFR clusters in normal cells (207 nm in diameter) were smaller than those in cancer cells (293 nm in diameter). They also performed simultaneous dSTORM imaging of EGFR and PALM imaging of PLCδ1-PH-mEOS3.2 in fixed COS-7 cells. Furthermore, dual-color PALM imaging by Raut et al. [75] showed the co-clustering of influenza A hemagglutinin and matrix protein M1 in the cell PM after chemical fixation. In all cases, PALM and dSTORM analyses after chemical fixation enabled the visualization of distinct and relatively large (>100 nm) lipid domains.

The lipid-binding protein is also applicable for detecting lipids other than membranes. Szracho et al. [76] reported, based on the simultaneous dual-color structured illumination microscopy (SIM) of fixed U2OS cells, that PI(4,5)P_2_ spatially orchestrates nuclear processes through its association with RNA and RNA-dependent PI(4,5)P_2_-binding proteins, thereby influencing their ability to form foci.

Koester et al. [103] developed a unique system to visualize the distribution of PS in fixed U2OS cells. They expressed GFP-fused Lact-C2, followed by fixation, saponin permeabilization, and incubation with an anti-GFP nanobody site-specifically conjugated to a single-stranded DNA oligonucleotide via site-specific click chemistry between AzPhe and dibenzocylooctyne (DBCO). The subsequent addition of fluorescently labeled single-stranded DNA, which transiently hybridized with the nanobody-conjugated DNA enabled DNA points accumulation for imaging in nanoscale topography (DNA-PAINT) at a spatial resolution of 20 nm. Dual-color DNA-PAINT analyses revealed that the constitutively active KRAS mutant G12V was almost invariably co-enriched with PS, while KRAS-G12D exhibited strong co-enrichment with PS in approximately half of the cells.

### 4.2. Observation in Living Cells

In living cells, super-resolution microscopy revealed markedly smaller lipid domains. For example, Ji et al. [77] expressed the photoactivable (PA)-mCherry-PH domains of PLCδ1 in INS-1 cells, and performed single-molecule imaging of PA-mCherry-PH of PLCδ1 at 50 ms/frame for PALM data acquisition. The PALM image demonstrated that the domain size of the PH of PLCδ1 in living cells was substantially smaller than that in the fixed cells.

**Figure 3:**
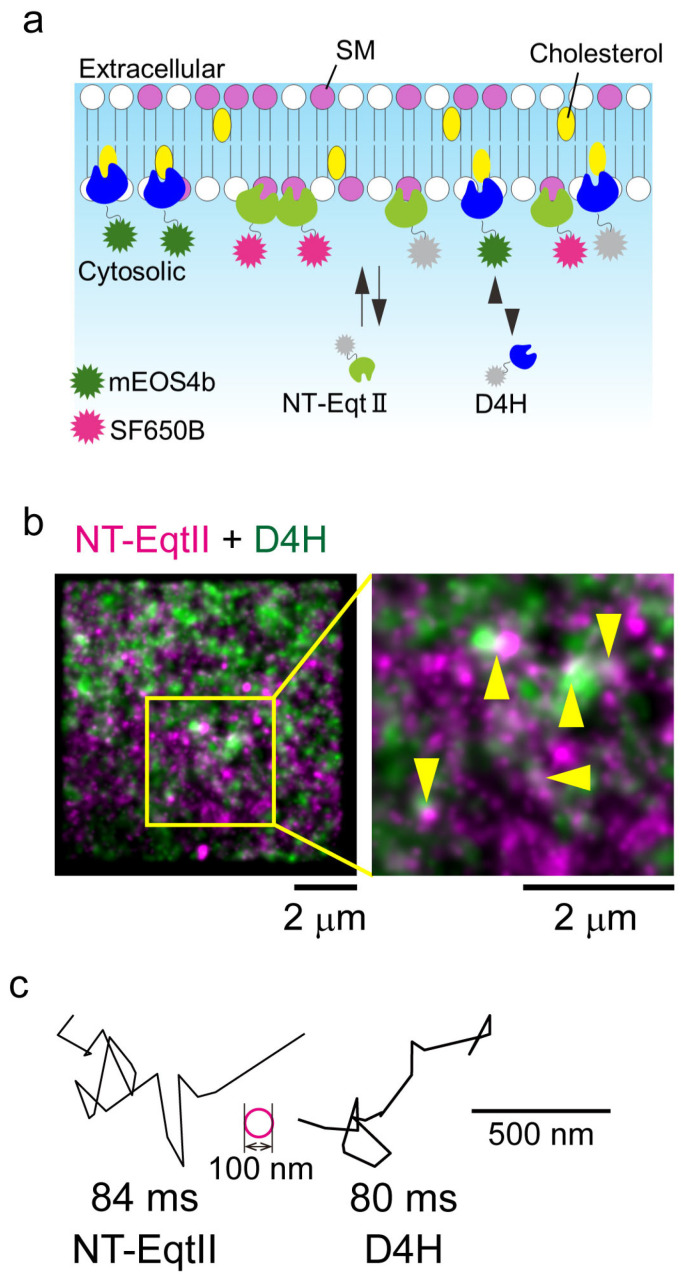
Small domain formation and rapid Brownian diffusion of lipids in the inner leaflet of cell PMs. (**a**). Schematic diagram of lipid-binding protein probes expressed in cells, which transiently bind to specific lipids in the inner leaflet of cell PMs. NT-EqtII and D4H are labeled with SF650B via Halo7-tag and with mEOS4b, respectively. (**b**). Dual-color super-resolution images of NT-EqtII-Halo7 labeled with SF650B (dSTORM, magenta) and D4H labeled with mEOS4b (PALM, green) in the inner leaflet of the COS-1 cell PM at 37 °C [65]. Colocalizations between the domains are indicated by yellow arrowheads. (**c**). Representative trajectories of single NT-EqtII and D4H molecules fused with tdStayGold in the COS-1 cell PM at 37 °C [65]. The scale bar indicates 500 nm. The magenta circle has a diameter of 100 nm. Adapted from Figure 2, Figure 6 and Figure 7 in [65] with permission.

As described above, Mori et al. [65] developed NT-EqtII for detecting SM in living cell membranes. Single-molecule imaging of NT-EqtII revealed the presence of SM in the inner leaflet of the PMs of 8 out of 15 cells in the steady state. They also performed super-resolution dSTORM imaging in living COS-1 cells, acquiring images by tracking single NT-EqtII molecules at 4 ms per frame for 2000 frames. ClusterViSu software (version 1.1.2) analysis [125] revealed that NT-EqtII clusters exhibited an average diameter of ~180 nm, comparable to that of D4H and Lyn-N20 (the N-terminal 20 amino acids of Lyn). Furthermore, the dual-color dSOTRM imaging of NT-EqtII combined with PALM imaging of D4H or Lyn-N20, followed by quantitative degree of colocalization (DoC) analysis [107], demonstrated that SM preferentially colocalized with cholesterol and Lyn-N20 in the inner leaflet of the living cell PM (Figure 3a,b). Collectively, these results strongly suggest the existence of raft-like domains in the inner leaflet of living cell PMs.

In living cells, the diffusional behaviors of lipid-binding proteins can also be investigated and analyzed at high temporal resolution. Mori et al. [65] performed single-molecule tracking of NT-EqtII, D4H, 2xPH of evectin-2, 2xPH of PLCδ, and Lyn-N20 fused with tdStayGold [126] at a 4 or 1 ms time resolution at 37 °C in the COS-1 cell PM. All of these molecular diffusions were classified as simple Brownian motion, rarely exhibiting temporal arrest of LateraL (TALL) diffusion longer than 32 ms in small domains of ~100 nm in diameter, and showed no immobilized diffusion (Figure 3c). These results indicate that these lipid-associated molecules are scarcely confined in small domains (<100 nm) for extended durations (>32 ms). Because the average compartment size constrained by transmembrane picket proteins along the actin-based membrane skeleton in COS-1 cells is 58 nm [44], with a residency time of less than 1 ms, temporal resolutions of 4 or 1 ms are insufficient to capture the hop diffusion of membrane molecules. This explains why all the observed molecules appeared to undergo simple Brownian diffusion. However, single-molecule imaging in the inner leaflet of living cell PMs currently remains unfeasible due to the lack of photostable fluorophores comparable to Cy3 for high-time-resolution observation. The future development of membrane-permeable, photostable organic dyes is expected to overcome this limitation.

The multi-color simultaneous single-molecule imaging of lipid-binding proteins and membrane molecules in living cells can elucidate their molecular associations. For example, by employing triple-color single-molecule imaging of a G-protein-coupled receptor, V2R, β-arrestin 2, and PI(4,5)P_2_ marker, PLCδ1-PH, Kuramoto et al. [78] demonstrated that ligand stimulation enhanced the colocalization of V2R and β-arrestin 2 with PLCδ1-PH, indicating that a noncanonical PI(4,5)P_2_ site is essential for the rapid recruitment of the V2R-β-arrestin2- PI(4,5)P_2_ complex into immobile membrane domains. In such imaging systems, the degree of colocalization between molecules is strongly influenced by the labeling efficiency of each probe, necessitating careful analytical consideration, including appropriate corrections.

## 5. Conclusions

In this review, we discussed lipid probes, including organic dye-conjugated lipids and lipid-binding proteins. Although these probes are highly valuable for visualizing lipid distribution and dynamics, enhancing their versatility requires improvements in their binding specificity, their cell toxicity, and the photostability of their fluorophores, as outlined herein. Moreover, as imaging technologies advance, lipid probes must be correspondingly refined. For example, a method of super-resolution live-cell movie imaging was recently established by the application of high-speed single-molecule observation techniques [24,25,127], with accompanying analytical tools now publicly available. Collectively, the continuous refinement of lipid probes in parallel with the evolution of imaging technologies will not only deepen our understanding of lipid organization and dynamics at the nanoscale in living cells but also open new avenues for elucidating their roles in diverse physiological and pathological processes.

## Figures and Tables

**Table 1:** Lipid probes conjugated with organic dyes for imaging, which are partitioned into Lo- or Ld-like phase.

Lipid	Probe	Fluorophore	Partitioning	References
Phosphoethanolamine (PE)	Cy3-DOPE	Cy3	-	[23]
Phosphoethanolamine (PE)	594-DOPE	ATTO594	Ld	[26]
Phosphoethanolamine (PE)	FITC-DOPE	Fluorescein (FITC)	-	[28]
Phosphoethanolamine (PE)	TMR-PE	TMR	-	[29]
Phosphoethanolamine (PE)	Atto647N-DPPE	ATTO647N	Ld	[30]
Phosphoethanolamine (PE)	DSPE-PEG-KK114	KK114	Lo	[31]
Phosphatidylcholine (PC)	594neg-DOPC	ATTO594	Ld	[26]
Phosphatidylcholine (PC)	594neg-DSPC	ATTO594	Lo	[26]
Phosphatidylcholine (PC)	BODIPY-FL-C5- or -C12-PC	BODIPY-FL	Ld	[32]
Phosphatidylcholine (PC)	β-BODIPY FLC12-HPC	BODIPY-FL	Ld	[33]
Sphingomylein (SM)	488neg-SM	ATTO488	Lo	[26]
Sphingomylein (SM)	594neg-SM	ATTO594	Lo	[26]
Sphingomylein (SM)	Atto647N SM	ATTO647N	Ld	[34]
GM1	488-S9-GM1	ATTO488	Lo	[27,35]
GM1	594-S9-GM1	ATTO594	Lo	[27,35]
GM1	BODIPY-FL-GM1	BODIPY-FL	Ld	[27]
GM1	Atto647N GM1	ATTO647N	Ld	[34]
GM1	Alexa-GM1	Alexa488, 568, 647	-	[36]
GM1	Alexa-peptide-GM1	Alexa-488	Lo	[37]
GM3	SF650B-GM3	SF650B	-	[24]
GM3	488-S9-GM3	ATTO488	Lo	[27,35]
GM3	594-S9-GM3	ATTO594	Lo	[27,35]
GM3	647N-S9-GM3	ATTO647N	Ld	[27]
GM3	FI-S9-GM3	Fluorescein (FI)	Lo	[27]
GM3	N9-FI-GM3	Fluorescein (FI)	Lo	[27]
Stage-specific embryonic antigen-3 (SSEA-3)	594-SSEA-3	ATTO594	Lo	[35,38]
Stage-specific embryonic antigen-4 (SSEA-4)	594-SSEA-4	ATTO594	Lo	[35,38]
Globohexaosylceramide (Globo-H)	594-Globo-H	ATTO594	Lo	[35,38]
Sialyl-lactotetraosylceramide (NeuAcLc4Cer)	ATTO594-NeuAcLc4-Cer	ATTO594	Lo	[35,39]
Lactotetraosylceramide (Lc4Cer)	ATTO594-Lc4Cer	ATTO594	Lo	[35,39]
GD2	ATTO594-GD2	ATTO594	Lo	[35,40]
GD3	ATTO594-GD3	ATTO594	Lo	[35,41]
GQ1b	ATTO594-GQ1b	ATTO594	Lo	[35,41]
Cholesterol	BODIPY-Chl 2	BODIPY	Lo	[42]
Cholesterol	Bdp-Chol	BODIPY	Lo	[43,44]
Cholesterol	Dehydroergosterol (DHE)	-	-	[45]
Cholesterol	Cholesterol analog 5	-	Lo/Ld	[46]

**Table 2:** Lipid-binding protein probes for imaging.

Lipid	Probe	Fluorophore	Subcellular Localization	References
PI(4,5)P_2_	PH domain of PLCδ1	FP */Halo/SNAP	PM inner leaflet	[61,62,65,66,67,68,69,70,71,72,73,74,75,76,77,78,79,80,81]
PI(4,5)P_2_	Tubby C-terminal domain	FP	PM inner leaflet	[70,82]
PI(3,4)P_2_	C-terminal PH domain of TAPP1	FP	PM inner leaflet	[71,79,80,83]
PI(3,5)P_2_	Dictyostelium SnxA	FP	PM inner leaflet	[84]
PI(3,5)P_2_	KIF1A PH domain	FP	Synaptic vesicle	[85]
PI(3,4,5)P_3_	PKB/Akt PH domain	FP/Halo	PM inner leaflet	[62,71,81,86,87,88]
PI(3,4,5)P_3_	GRP1 PH domain	FP	PM inner leaflet	[63,67,89]
PI(3,4,5)P_3_	Btk1 PH	FP	PM inner leaflet	[62,79,80,90,91]
PI(4)P	FAPP1	FP	Golgi, PM	[72,92]
PI(4)P	P4M domain (SidM/SidC)	FP	Golgi, PM	[71,72,80]
PI(3)P	EEA1 FYVE domain	FP	Endocytic compartment	[71,72,93,94,95,96]
PI(3)P	Hrs 2xFYVE domain	FP	Early endosome	[79,80,84,93,97]
Phosphatidylserine (PS)	Lactadherin C2 (Lact-C2)	FP/Halo	PM inner leaflet	[66,73,81,98,99,100,101,102,103,104,105]
Phosphatidylserine (PS)	Evectin-2-PH domain	FP	Endosomes	[65,67,102,106]
Cholesterol	D4H (domain 4 of perfringolysin O)	FP	PM inner leaflet	[65,73,81,107,108,109]
Sphingomylein (SM)	NT-lysenin (C-terminal residues 161–297)	FP	PM outer leaflet	[66,110,111,112]
Sphingomylein (SM)	W20A mutant	FP	PM outer leaflet	[66,113]
Sphingomylein (SM)	NT-EqtII (EqtII mutant)	FP/Halo	PM outer and inner leaflet	[65]

* FP; fluorescent protein.

## Data Availability

No new data were created or analyzed in this study. Data sharing is not applicable to this article.
